# Evaluation of an Advanced Topics Workshop in Geropsychology: The Value of Depth in Training

**DOI:** 10.1093/geroni/igab046.108

**Published:** 2021-12-17

**Authors:** Jeffrey Gregg, Rachel Rodriguez, Priyanka Mehta, Christine Gould

**Affiliations:** 1 Durham VA Health Care System, Durham, North Carolina, United States; 2 VA Palo Alto GRECC, Palo Alto, California, United States; 3 VA Palo Alto Health Care System, Palo Alto, California, United States

## Abstract

The Geriatric Scholars Program- Psychology Track (GSP-P) was implemented to address the dire shortage of mental health providers with geriatrics expertise within the VA, a large integrated healthcare system. One hundred and five psychologists participated in the GSP-P introductory geropsychology competencies course. Though they exhibited significant improvements in confidence, knowledge, and skills across geropsychology domains, increased depth (in addition to breadth) of training is needed. In 2019, GSP-P implemented an advanced workshop for graduates of the introductory course. Twenty-one psychologists participated in the workshop, which included 3.5 days of expert-led seminars followed by completion of an individualized learning plan over six months. Results from our evaluation indicated significant improvements in four of five geropsychology domains on the Pikes Peak Geropsychology Knowledge and Skill Assessment Tool. Our findings demonstrate continued enhancement of geropsychology competencies through advanced coursework is feasible and improves depth of training, particularly when combined with individualized learning plans.

